# Research on short-term photovoltaic power generation forecasting model based on multi-strategy improved squirrel search algorithm and support vector machine

**DOI:** 10.1038/s41598-024-65159-1

**Published:** 2024-06-21

**Authors:** Ruijin Zhu, Tingyu Li, Bo Tang

**Affiliations:** 1 Electric Engineering College, Tibet Agriculture and Husbandry College, Nyingchi, 860000 China; 2 Daqing Power Supply Company of State Grid Heilongjiang Electric Power Co., Ltd, China, DaQing, 163000 China

**Keywords:** PV power generation forecasting, Support vector machine regression, Squirrel search algorithm, Multi strategy improvement method, Principal component analysis, Electrical and electronic engineering, Photovoltaics

## Abstract

Solar photovoltaic (PV) power generation is susceptible to environmental factors, and redundant features can disrupt prediction accuracy. To achieve rapid and accurate online prediction, we propose a method that combines Principal Component Analysis (PCA) with a multi-strategy improved Squirrel Search Algorithm (SSA) to optimize Support Vector Machine (MISSA-SVM) for prediction. Initially, to mitigate the impact of redundant features on prediction accuracy, KPCA is employed for feature dimensionality reduction. Subsequently, SVM is suggested as the foundational algorithm for constructing the prediction model. Furthermore, to address the influence of hyperparameter selection on model performance, SSA is introduced for optimizing SVM hyperparameters, with the aim of establishing the optimal prediction model. Moreover, to enhance solution efficiency and accuracy, a multi-strategy approach termed MISSA is proposed, which integrates Population Initialization based on the Tent map, Nonlinear Predator Presence Probability, Chaotic-based Dynamic Opposition-based Learning, and Selection Strategy, to refine SSA. Finally, through case studies, the performance of MISSA optimization is assessed using challenging CEC2021 test functions, demonstrating its high optimization performance, stability, and significance. Subsequently, the performance of the prediction model is validated using two datasets, showcasing that the proposed prediction method achieves high accuracy and robust prediction stability.

## Introduction

When large-scale photovoltaic (PV) power stations are connected to the power grid, it will have a serious impact on the security and stability of the power system^1,2^. Therefore, it is of great significance to predict the power generation of PV power stations quickly and accurately^3^.

At present, photovoltaic power generation forecasting methods mainly include direct method and indirect method^4^.

The indirect method obtains the required solar irradiance, rainfall, wind speed, humidity, temperature and other data according to the weather forecast given by the meteorological station, establishes a model in combination with the installation angle, installation position, conversion efficiency and other parameters of the photovoltaic panel, and directly obtains the corresponding time corresponding photovoltaic power generation through the calculation formula^5^. Ma et al. proposed A simulation model for modeling photovoltaic (PV) system power generation and performance prediction, compared with other models in the simulation performance, and further validated by outdoor tests, the advantage of this model is that the model does not need a lot of historical data for training, but the model relies on the detailed design data of photovoltaic power station and photoelectric conversion parameters, and its robustness is poor^6^. At the same time, the indirect method is greatly affected by the weather data. When the weather does not suddenly change dramatically, the method can obtain more accurate prediction results; When the weather changes suddenly, the method will be affected, thus reducing the prediction accuracy^7^.

The direct method includes statistical prediction method and artificial intelligence prediction method. The statistical prediction method conducts curve fitting according to historical data such as weather and solar radiation to establish the mapping model of input and output and realize the prediction of photovoltaic power generation^8^. Common statistical prediction methods include time series^7,9^, regression analysis^10^, fuzzy theory^11^ and grey theory^12^. The generalized ARMAX time series model was proposed by Li et al. to predict photovoltaic power, the simulation results are shown that the ARMAX model greatly improves the forecast accuracy of power output^9^. To forecast solar power generation, Eungeun et al. proposed a fuzzy clustered FL algorithm (FCFLA) and achieved better results that this method had higher predict accuracy and fastest convergence time^11^. The statistical prediction method does not need to consider the conversion efficiency of photovoltaic arrays and the installation angle of photovoltaic panels, so this method has the advantage of simple modeling compared with the physical method. However, most of the statistical prediction methods are linear prediction, which is not conducive to long-term and large-scale photovoltaic system power generation prediction. The prediction is difficult, and the model relies on a large number of historical valid data, so the prediction effect is average.

To sum up, both indirect method and statistical prediction method have some defects; therefore, researchers are focusing more on PV power generation prediction based on artificial intelligence prediction methods. At present, the main statistical methods include neural networks (NNs)^13^, SVM^14^, deep learning^15–17^.

Reference^15^ integrates deep learning principles and conducts an in-depth study based on the extreme gradient boosting algorithm combined with Artificial Neural Networks (ANN) and Long Short-Term Memory (LSTM). They propose an improved generally applicable stacked ensemble algorithm (DSE-XGB) and conduct research on model interpretability, ultimately achieving promising experimental results. In reference^16^, a physical problem and a deep learning model are proposed for predicting photovoltaic power generation. The authors conduct research based on Long Short-Term Memory (LSTM) and integrate physical constraints to enhance the rationality and interpretability of predictions. They analyze the predictive capability using real datasets and demonstrate through simulations that the proposed method effectively improves prediction accuracy, exhibiting strong superiority. In Ref.^17^, a new hybrid deep residual learning and gated Long Short-Term Memory recurrent network is proposed based on deep learning. The authors develop a comprehensive deep learning framework and compare it with the proposed method. The results indicate that a cooperative architecture of gated recurrent units (GRU) and Long Short-Term Memory (LSTM) recurrent models can enhance the performance of Xception and ResNet. Li et al. proposed a power generation forecasting model for PV power stations based on the combination of principal component analysis (PCA) and backpropagation NNs (BPNNs); the examples in their paper show that the method proposed by the authors have high prediction accuracy. However, the prediction performance of BPNN models depends on the parameter settings, and the authors have not used other methods to optimize BPNNs^18^.

Ullah et al. dive deep into forecasting methods, analyze their performance, and finally, present a novel three-tier framework for ECP, and the results show that this methods have the best prediction ability^19^. Later, Ullah's team proposed a new method based on Deep Convolutional LSTM and Stacked GRU to extract of deep knowledge from complex energy data, and the experiment proved that the proposed method revealed promising results^20^. Zhen et al. proposed a novel ultra-short-term PV power prediction model based on the improved bidirectional long short-term memory model with a genetic algorithm (GA-BLSTM). The prediction model described in their paper has strong advantages. However, the method proposed by the authors have not been compared with the prediction model in the literature in recent years, and the genetic algorithm (GA) also has some defects, such as its tendency to fall into local optimization^21^. Pan et al. explored data pre-processing based on an ultra-short-term PV model using improved ant colony optimization to enhance SVM. The results indicate that their proposed method is better. However, the authors could only compare the traditional models and could not prove the validity of the proposed model^22^. Meng et al. proposed a forecasting model based on a random forest algorithm. The case study shows that the model proposed by the authors are superior to the other three models, but the authors have not made any improvements to the random forest algorithm, and the prediction performance of the traditional random forest algorithm still needs to be strengthened^23^.

Analyzing the above literature and comprehensively considering the less power generation data used is. And according to literature^24^, extensive research has been conducted on neural network methods, and the findings suggest that traditional deep neural networks typically require a substantial amount of data for effective training. Consequently, neural networks may not be well-suited for the small sample dataset examined in this paper. So SVM is selected as the prediction model, which is suitable for processing small samples and nonlinear data^25,26^. However, with SVM, it is difficult to determine the super parameters, which can lead to poor performance. Therefore, to establish the optimal prediction model, experts and scholars have extracted various methods to improve the prediction performance of SVM^27–30^.

Zhang et al. achieved good results using particle swarm optimization (PSO) to optimize SVM. However, the population diversity of the PSO is poor, and the optimization performance is easily affected by these parameters^31^. Wang et al. proposed a prediction model based on environmental factors and an SVM optimized by a GA (GA-SVM), and the results show that the proposed model is effective. However, the authors did not study the defects of the GA, such as its tendency to fall into local optimization and its slow convergence speed^32^. William et al. Considered the influence of stochastic weather conditions on the output power of PV systems, proposed a short-term PV power forecasting based on GASVM model, and achieved good simulation results. However, authors did not study the defect that GA can easily fall into local optimization, and did not compare it with other common prediction methods^33^. Li et al. proposed an improved sparrow search algorithm to optimize SVM and construct the mid-long-term load prediction model, and the results show that the improved sparrow search algorithm-SVM has a better prediction performance. The authors had not improved the initial population and updated formula of the sparrow search algorithm, and the improved method cannot significantly improve the optimization performance of sparrow search algorithm^34^.

In summation, a methodology is introduced that combines PCA with MISSA-SVM prediction model. In order to enhance diagnostic efficiency and reduce the influence of unimportant features on predictions, PCA is employed for feature dimensionality reduction. Simultaneously, to enhance the diagnostic performance of the SVM model and address the impact of hyperparameters on model performance, a multi-strategy improved SSA, based on Population Initialization using the Tent map, Nonlinear Predator Presence Probability, Chaotic-based Dynamic Opposition-based Learning, and Selection Strategy, is proposed for optimizing SVM hyperparameters. It is noteworthy that the proposed model is not suitable for the field of photovoltaic forecasting with large datasets.

The contributions made in this paper are as follows. First, PCA is used to analyze input data and reduce the influence of the principal component with low contribution rates of prediction accuracy. To build the optimal model, this paper then proposes an improved squirrel search algorithm (SSA) based on a multi-strategy, which uses a tent map to improve the diversity of the squirrel population. The nonlinear *P*_*dp*_ is used to increase the optimization ability of the squirrel population in the latter stage, and chaotic map-based dynamic opposition-based learning (OBL) and a selection strategy for differential evolution (DE) are used to improve the convergence speed and optimization accuracy of the SSA. In addition, benchmark test functions are used to test the optimization performance of the MISSA and the other four typical metaheuristic algorithms. The results show that the MISSA has the best optimization performance. Finally, using the data of two typical months, the proposed model is compared with the other four prediction models. The results show that the root mean square error (RMSE), mean square error (MSE), and mean absolute error (MAE) of the proposed model are superior, and the corresponding fitness curve is also superior, proving that the accuracy and effectiveness of the proposed prediction model are strong.

The main structure of this paper is as follows: “Support vector machine” introduces the basic theory of SVM; “Improved squirrel search algorithm” presents the basic theory of SSA and its improvement strategies, and tests the optimization performance of MISSA; “Prediction model of PV power generation based on MISSA-SVM” establishes a photovoltaic power generation prediction model based on MISSA-SVM, and introduces the prediction process; “Prediction of PV power generation based on MISSA-SVM” uses two simulation examples for analysis; “Conclusion” concludes the entire article and provides future prospects.

## Support vector machine

The basic principle of an SVM is to map the sample data to high-dimensional space through nonlinear mapping and then carry out linear regression in high-dimensional space^35^. The basic model is as follows:

1 $$f(x) = {\omega^T}x + b$$

When performing regression analysis, the SVM allows for a deviation, *ε*, between* f* (*x*) and *y*:

2 $$\mathop {\min }\limits_{\omega ,b} \frac{1}{2}||\omega |{|^2} + C\sum\limits_{i = 1}^m {l_\varepsilon } \left( {{\text{f}}\left( {x_i} \right) - {y_i}} \right)$$

where C is the regularization constant and *l*_*ε*_ is the insensitive loss function. Introducing relaxation variables *ξ* and Lagrange multipliers can be gave:

3 $$\begin{aligned} & L\left( {\omega ,b,\alpha ,\hat \alpha ,\xi ,\hat \xi ,\mu ,\hat \mu } \right) = \frac{1}{2}||\omega |{|^2} + C\sum\limits_{i = 1}^m {\left( {{\xi_i} + {{\hat \xi }_i}} \right)} - \sum\limits_{i = 1}^m {\mu_i} {\xi_i} - \sum\limits_{i = 1}^m {{{\hat \mu }_i}} {{\hat \xi }_i} \\ & \quad + \sum\limits_{i = 1}^m {\alpha_i} \left( {f\left( {x_i} \right) - {y_i} - \varepsilon - {\xi_i}} \right) + \sum\limits_{i = 1}^m {{{\hat \alpha }_i}} \left( {{y_i} - f\left( {x_i} \right) - \varepsilon - {{\hat \xi }_i}} \right) \\ \end{aligned}$$

where the Lagrange multipliers $\alpha ,\hat \alpha ,\mu ,\hat \mu \geqslant$ 0 let the partial derivative of the Lagrange function be $\omega ,b,\xi ,\hat \xi$ = 0, and the original problem is transformed into a dual problem that meets the KKT condition. After inputting Eq. (1), the objective function of the SVM regression is:

4 $$f(x) = \sum\limits_{i = 1}^m {\left( {{{\hat \alpha }_i} - {\alpha_i}} \right)} \kappa \left( {x,{x_i}} \right) + b$$

where $K({x_i},{x_j})$ is the kernel function. The radial basis function has only one parameter, excellent generalization ability, and good performance in processing nonlinear data:

5 $$K({x_i},{x_j}) = \exp \left\{ { - \frac{{{{\left\| {{x_i} - {x_j}} \right\|}^2}}}{{2{\sigma^2}}}} \right\}$$

where *σ* is the kernel function parameter. To find the optimal hyperparameters (*σ* and* C*) of SVM, MISSA is proposed in this paper.

## Improved squirrel search algorithm

SSA is an optimization algorithm based on squirrel foraging behavior proposed by Mohit Jain et al. in 2017^36,37^. Because SSA has some basic research, this paper will not repeat its basic theory. At the same time, after analyzing its principle and optimization process, in this paper, a strategic method to improve the shortcomings of SSA is proposed.

### Improved method based on squirrel search algorithm

Compared with traditional optimization algorithms, the SSA has better optimization performance, but it still has problems with poor population diversity and a tendency to fall into local optimization. Therefore, this section proposes three methods to improve the SSA.

#### Population initialization based on Tent map

In view of the poor diversity of the initial population of squirrels, Tent map is used to initialize the population. Tent map has the characteristics of strong ergodicity and randomness^38^. The specific formula is as follows:

6 $${x_{n + 1}} = \left\{ {\begin{array}{*{20}{c}} {2{x_n},{x_n} \in [0,0.5)} \\ {2(1 - {x_n}),{x_n} \in [0.5,1)} \end{array}} \right.$$

**Figure 1:**
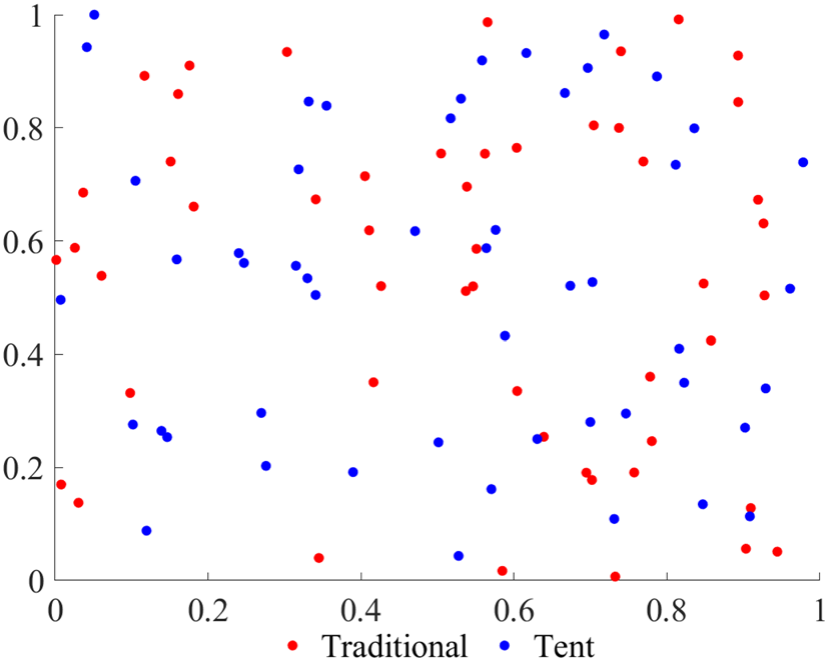
Comparison of initial population.

Figure 1 shows the initial population generated by two population initialization methods:

Figure 1 shows that, compared with the original population initialization method, the population ergodicity and randomness mapped by the tent map are higher, which will improve the diversity of the initial squirrel population.

#### Nonlinear predator presence probability

**Figure 2:**
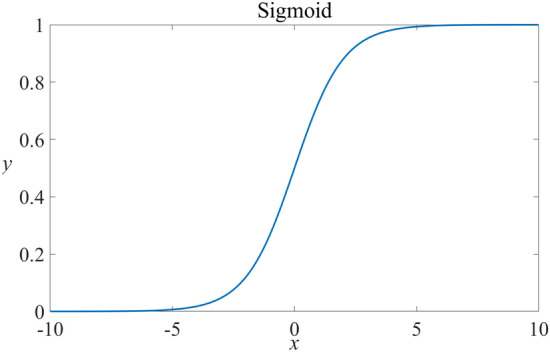
Sigmoid function curve.

Addressing the defects of *P*_*dp*_ can give the squirrel population the probability to jump out of local optimization and conduct global optimization again at a later stage. Based on the sigmoid function, this paper proposes a probability formula of nonlinear attenuation to balance the optimization behavior of squirrels and improve the optimization performance. The sigmoid function is as follows^39^, and the related image is shown in Fig. 2:

7 $$f(x) = \frac{1}{{\left( {1 + {e^{ - x}}} \right)}}$$

**Figure 3:**
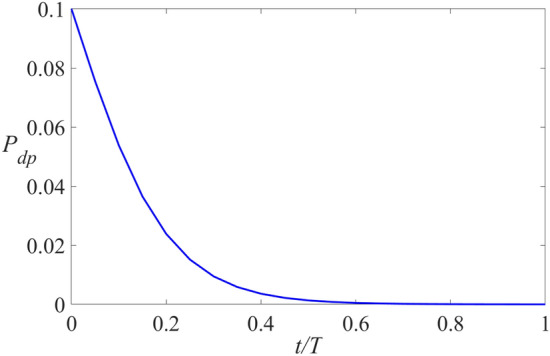
*P*_*dp*_ curve.

The improved formula based on the sigmoid function is as follows, and the specific function image is shown in Fig. 3.

8 $${P_{dp}} = \frac{2}{{\left( {1 + {e^{10 \cdot \frac{t}{T}}}} \right) \times 10}}$$

In Fig. 3, *P*_*pd*_ will decay nonlinearly, rapidly approaching 0 from 0.1; *t* is the current iteration number, and *T* is the maximum iteration number. This method can effectively improve the foraging ability of the squirrel population and reduce the probability of random foraging.

#### Chaotic based dynamic opposition-based learning

**Figure 4:**
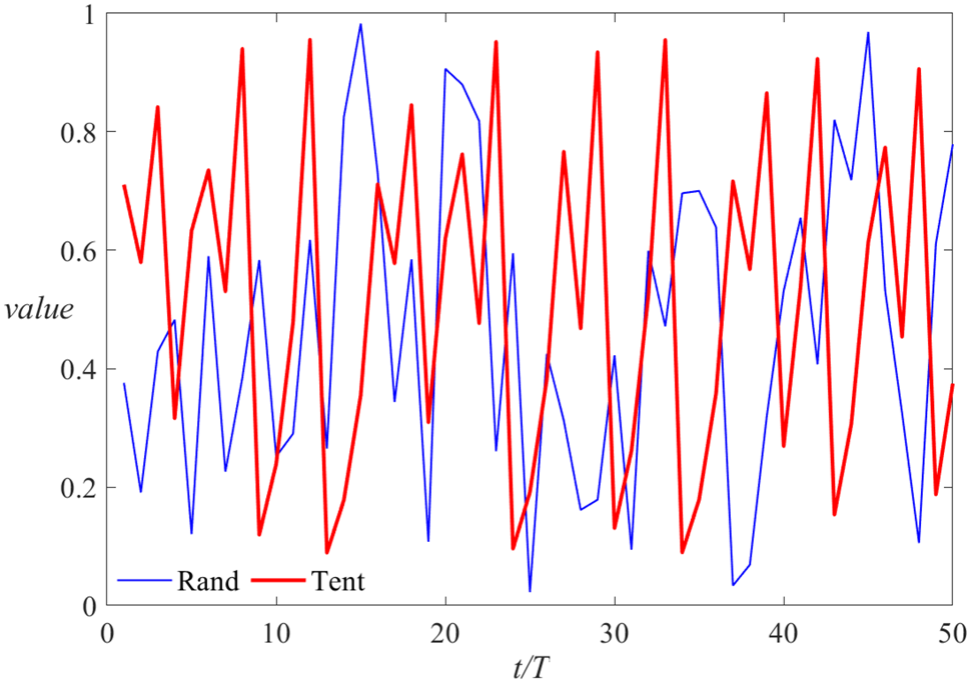
Comparison of random sequences.

Aiming at the squirrel population iteration’s tendency to fall into local optimization, after the position update, this paper proposes a chaotic based dynamic OBL to update the squirrel position. The OBL is as follows:

9 $$x_i^{t + 1} = (ub + lb) - x_i^t$$

where *ub* and *lb* are the upper and lower bounds of the population, respectively. On this basis, and considering the elite OBL^23^, this paper proposes a chaotic based dynamic OBL. The specific formula is as follows:

10 $$FS_{nt}^{dyol} = Tent \cdot (dyub + dylb) - FS_{nt}^{new}$$

where *dy*(*ub*)and *dy*(*lb*) are the upper and lower dynamic limits respectively, which are mainly determined by the maximum and minimum values of the squirrel population in its current iteration. At the same time, using a Tent map replaces the random number generated by R and increases the randomness of the position update. Figure 4 shows the random sequence generated by two methods in 50 iterations.

As shown in Fig. 4, the sequence generated by the Tent map has higher randomness and ergodicity.

#### Selection strategy

After the dynamic OBL, the selection strategy of DE is used to select the optimal location of the squirrel population.

11 $$FS_{nt}^{new} = \left\{ {\begin{array}{*{20}{c}} {FS_{nt}^{new}} \\ {FS_{nt}^{dyol}} \end{array}} \right. \, , \, \begin{array}{*{20}{c}} {f(FS_{nt}^{new}) < f(FS_{nt}^{dyol})} \\ {f(FS_{nt}^{new}) \geqslant f(FS_{nt}^{dyol})} \end{array}$$

where *FS*^*new*^ is the solution generated by the original population renewal formula, *FS*^*dyol*^ is the solution generated based on dynamic reverse learning, and *f*(*FS*^*new*^) and *f*(*FS*^*dyol*^) are fitness values. After the dynamic OBL increases the optimization performance of the squirrel population, the optimal population in the iteration is maintained by a selection strategy.

### Multi-strategy improved squirrel search algorithm summary

This paper uses the proposed method to improve the SSA and achieve MISSA to improve the performance of the optimization.

1. Improved method (1)—population initialization based on a Tent map: The original random initialization of SSA is replaced by Eq. (6), and the diversity of the squirrel population generated based on tent initialization is increased.
2. Improved method (2)—nonlinear predator presence probability: *P*_*dp*_ based on the sigmoid function, such as Eq. (8), is proposed. Compared with the traditional *P*_*pd*_, the proposed formula can quickly approach 0 from 0.1 for nonlinear attenuation to increase the optimization ability of the SSA in the middle and late stages.
3. Improved method (3)—chaotic based dynamic OBL: A chaotic dynamic OBL is proposed to obtain the reverse solution of the squirrel population before the end of the current iteration, which will greatly improve the optimization performance of the SSA.
4. Improved method (4)—selection strategy: A selection strategy based on DE is proposed to select the optimal solution from the reverse solution and the current solution for assignment to maximize the advantages of the population and improve the optimization performance of the population again.

The optimization process of MISSA will be represented by a pseudo code, as shown in Algorithm 1. At the same time, in order to verify the optimization performance of MISSA, it should be compared with the other four metaheuristic algorithms.
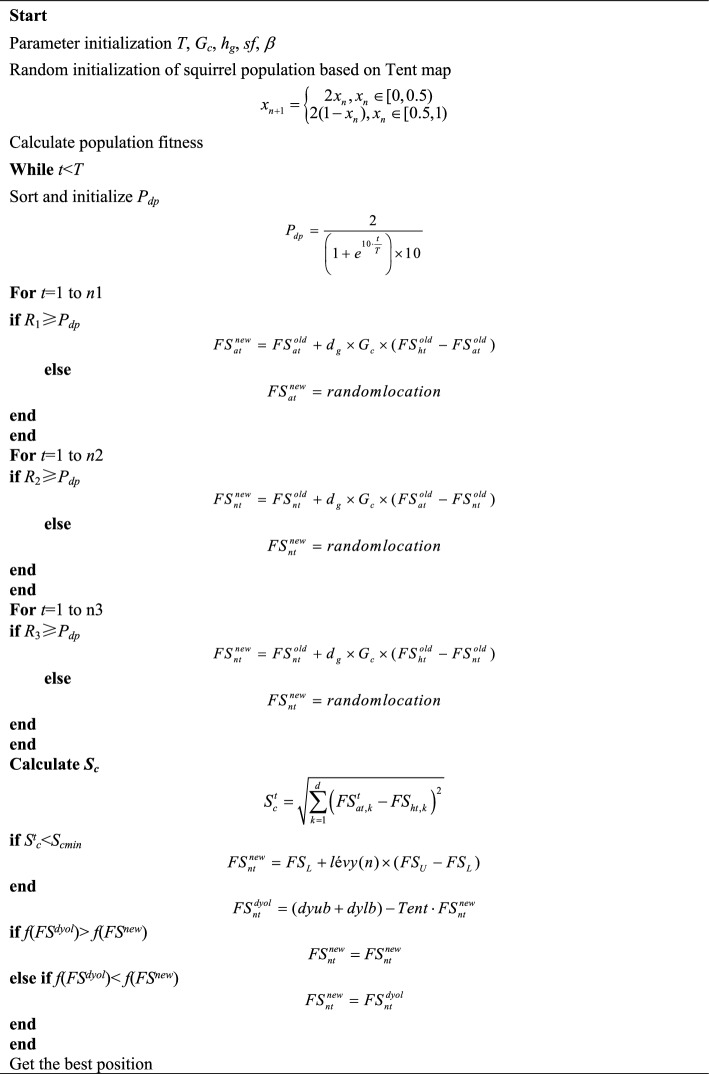


### Optimization performance test based on CEC2021

**Figure 5:**
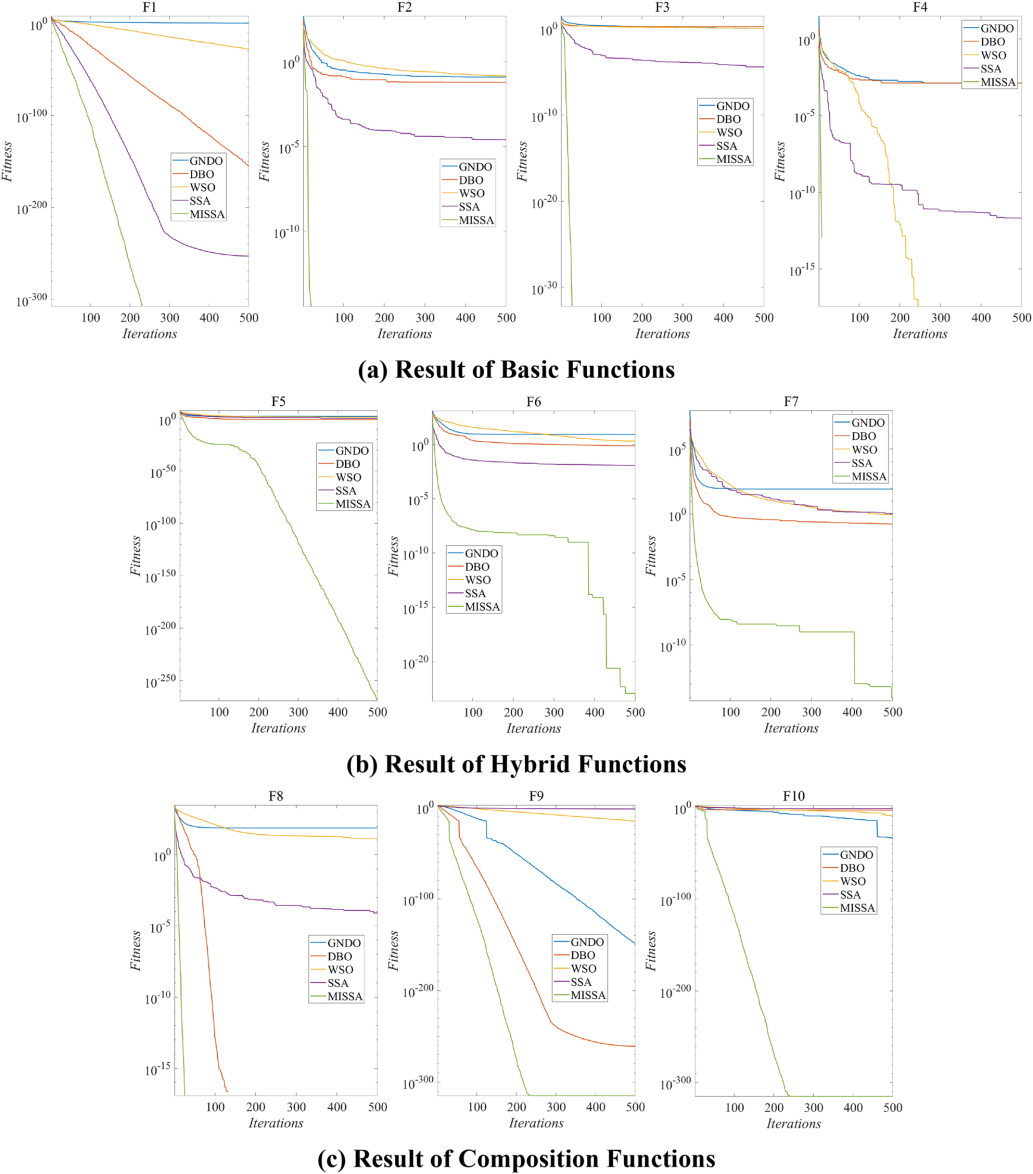
Test results of CEC2021.

This paper presents the optimization performance of the proposed optimization algorithm on challenging CEC2021 benchmark test functions and compares it with the optimization results of GNDO, DBO, WSO, and SSA. The effectiveness of the proposed algorithm is evaluated using CEC2021 benchmark test functions, which comprehensively test the optimization performance on Basic functions, Hybrid Functions, and Composition Functions sets. The parameter settings for all algorithms are provided in Table 1. Additionally, since the SVM hyperparameter optimization problem is a two-dimensional optimization problem, the dimensionality of all test functions is set to 2. Each optimization algorithm is run 30 times on each test function, and the results are statistically summarized using three metrics: best value, average value, and standard deviation. The CEC2021 functions are introduced in Table 2, and the average best fitness curves are illustrated in Fig. 5 and summarized in Table 3.

**Table 1:** Parameters of algorithms.

Algorithms	Parameters	Value
GNDO	*a*_1_, *a*_2_	2, 1
	*GP*	0.5
DBO	*δ*	0.9
WSO	*P*_*max*_	1.5
	*P*_*min*_	0.5
SSA	*G*_*c*_	1.9
	*P*_*dp*_	0.1
MISSA	*P*_*dp*_	Equation (8)
Common	*M*	50
	*MaxIter*	500

**Table 2:** CEC2021 benchmark test functions.

Function set	Function name	Dim	Range	*f*_*min*_
Basic functions	Bent Cigar Function	30	[− 100, 100]	0
	Shifted and Rotated Schwefel’s Function	30	[− 100, 100]	0
	Shifted and Rotated Lunacek bi-Rastrigin Function	30	[− 100, 100]	0
	Expanded Rosenbrock’s plus Griewangk’s Function	30	[− 100, 100]	0
Hybrid Functions	Hybrid Function 1	30	[− 100, 100]	0
	Hybrid Function 2	30	[− 100, 100]	0
	Hybrid Function 3	30	[− 100, 100]	
Composition Functions	Composition Function 1	30	[− 100, 100]	
	Composition Function 2	30	[− 100, 100]	
	Composition Function 3	30	[− 100, 100]

**Table 3:** Test results of CEC2021.

Functions	GNDO			DBO			WSO			SSA			MISSA		
	Best	Mean	Std	Best	Mean	Std	Best	Mean	Std	Best	Mean	Std	Best	Mean	Std
F1	2.1e−06	0.71	1.28	3e−163	5e−156	2e−155	9.7e−32	3.1e−28	8.7e−28	1e−310	1e−253	**0**	**0**	**0**	**0**
F2	1.1–13	0.12	0.19	**0**	0.061	0.16	2.9e−03	0.14	0.14	2.2e−09	2.5e−05	4.7e−05	**0**	**0**	**0**
F3	1.9e−31	1.05	0.91	**0**	1.64	0.83	0.013	1.06	0.74	3.1e−08	3.5e−05	5.8e−05	**0**	**0**	**0**
F4	1.1e−16	1.3e−03	5.1e−03	**0**	1.3e−03	4.2e−03	**0**	**0**	**0**	**0**	2.1e−12	7.4e−12	**0**	**0**	**0**
F5	1.2	2e + 02	2.1e + 02	1.8e−30	0.15	0.42	1.21	29.3	53.6	1.5e−02	1.83	3.26	**0**	**5.e**−**270**	**0**
F6	0.17	8.15	39.6	2.4e−02	0.71	1.19	0.68	1.99	2.31	1.8e−03	1.1e−02	6.2e−03	**0**	**2.3e**−**24**	**1.3e**−**23**
F7	0.15	84.4	113.1	4.9e−05	0.17	0.49	8.3e−02	0.86	1.63	3.3e−03	1.12	1.74	0	5.4e−15	2.2e−14
F8	1.1e−10	71.7	63.9	**0**	**0**	**0**	8.6e−08	13.1	11.4	2.6e−07	7.5e−05	2.4e−04	**0**	**0**	**0**
F9	9e−162	4e−149	2e−148	**1e**−**315**	1e−261	**0**	1.4e−37	5.9e−16	2.2e−15	4.2e−05	7.6e−03	8.3e−03	**1e**−**315**	**1e**−**315**	**0**
F10	8.9e−57	1.1e−15	4.6e−15	9.1e−05	5.2e−04	2.4e−04	3.8e−11	1.5e−07	3.1e−07	7.8e−03	2.9e−02	1.3e−02	**0**	**1e**−**315**	**0**

Significant values are in bold.

The optimization performance of GNDO, DBO, WSO, SSA, and MISSA was tested and compared. In the Basic Functions, MISSA consistently found the minimum value of the functions with high efficiency. Analyzing the results of the Hybrid Functions optimization, several advanced algorithms tended to get trapped in local optima with lower precision, while MISSA demonstrated sustained performance and higher optimization accuracy. For the Composition Functions optimization, MISSA maintained fast optimization efficiency while ensuring high precision in solutions. These results effectively demonstrate the advantages of the proposed optimization method. To further validate its superiority and stability, the evaluation metrics including Best, Mean, and Std were used to summarize the results of 30 tests, as shown in Table 3.

In Table 3, all optimal metrics are highlighted in bold font. In the Basic Functions, MISSA consistently found the minimum value of the functions in all 30 runs, demonstrating stable optimization performance. Analyzing the results of the Hybrid Functions optimization, MISSA achieved the minimum value with significantly better average and standard deviation compared to other algorithms. Regarding the Composition Functions optimization, MISSA consistently found the minimum values for F8 and F10, while for F9, DBO and MISSA achieved the same optimal value, but MISSA exhibited extremely high stability in its solutions.

The above analysis compares MISSA with four other advanced optimization algorithms in solving the CEC2021 test functions, effectively demonstrating the superior optimization performance of MISSA. Furthermore, to validate the significance of MISSA, we conducted Wilcoxon tests.

Wilcoxon is a statistical learning method used to test the significance of models, with its primary parameter being the p-value. For example, considering the entry at row 2, column 2 of the Table 4 (p-value equals 1.2118e−12), this implies that p < 0.05. Thus, compared to GNDO, MISSA exhibits significant differences. On the other hand, if p ≥ 0.05, the opposite result would occur, as seen in row five, column four of the Table 4. In conclusion, it can be inferred that MISSA demonstrates strong significance.

**Table 4:** Test results of Wilcoxon.

Function	GNDO	DBO	WSO	SSA
F1	1.2118e−12	1.2118e−12	1.2118e−12	1.2118e−12
F2	1.0662e−12	4.1926e−2	1.2118e−12	1.2118e−12
F3	1.1575e−12	1.9457e−09	1.2118e−12	1.2118e−12
F4	4.1574e−14	1.2118e−12	NaN	1.9332e−10
F5	2.8646e−11	2.8646e−11	2.8646e−11	2.8646e−11
F6	1.7203e−12	1.7203e−12	1.7203e−12	1.7203e−12
F7	2.2623e−11	2.2623e−11	2.2623e−11	2.2623e−11
F8	1.2118e−12	NaN	1.2118e−12	1.2118e−12
F9	3.0199e−11	6.6955e−11	3.0199e−11	3.0199e−11
F10	3.0199e−11	3.0199e−11	3.0199e−11	3.0199e−11

## Prediction model of PV power generation based on MISSA-SVM

This section will specifically introduce the prediction model of PV power generation based on MISSA-SVM.

### Data pre-processing

The data in this paper comes from the power generation data of a 23.4 kW PV power station between the times of 8 a.m. and 5 p.m.^33^. Additionally, for the effectiveness of the experiment, two typical data sets were used from the months of June and December.

The number of data characteristics is shown in Table 5, and the data from June and December are shown in Tables 6 and 7, respectively.

**Table 5:** Number of data characteristics.

Number	Characteristic
1	Wind speed
2	Weather temperature celsius
3	Weather relative humidity
4	Global horizontal radiation
5	Diffuse horizontal radiation
6	Wind direction

**Table 6:** Data from June.

Num	1	2	3	4	5	6	Active power
1	2.909	11.961	68.785	97.325	77.413	115.816	2.779
2	3.098	12.639	65.729	148.864	139.965	99.987	3.830
3	4.868	13.664	61.903	420.433	209.613	115.803	11.369
4	4.964	14.977	55.284	498.893	218.004	109.526	12.019
5	4.059	16.317	50.948	715.889	95.901	139.850	16.578
6	3.483	17.175	47.941	703.717	99.465	124.719	18.472
7	3.837	18.015	45.716	628.280	70.499	128.149	19.108

**Table 7:** Data from december.

Num	1	2	3	4	5	6	Active power
1	2.967	33.261	25.216	1099.837	102.280	111.695	16.869
2	3.268	34.756	21.328	1027.314	112.693	157.851	16.619
3	4.246	34.720	20.353	872.337	108.730	126.718	16.836
4	4.188	34.409	19.619	640.870	88.692	122.827	15.671
5	4.946	33.873	18.651	416.405	78.393	136.552	13.826
6	4.799	22.442	26.059	431.153	158.176	124.502	11.666
7	4.112	24.383	23.904	794.835	102.652	96.242	17.318

The dataset is set up with sliding windows. Assuming the original data size is *m* × *n*, and the length of the sliding window is set to *l*, the entire rectangular sliding window size would be *l* × *n*. Typically, the sliding step size is set to 1, resulting in (*m *− *l* + 1) rectangular datasets after sliding window processing. This study involves two datasets, each spanning 30 days. Each day's data consists of 10 entries with 6 features, making the total data size 300 × 6. Based on experimentation, it was found that a sliding window size of 70, equivalent to 7 days, yields the best prediction performance. Hence, the sliding window size obtained is 70 × 6, resulting in a final set of 24 rectangular datasets, each sized 20 × 6.

To reduce the complexity of the data and eliminate the feature quantities with low correlations, PCA is used to process the two data sets. The specific results are shown in Table 8, where CR represents the principal component contribution rate and TCR represents the total contribution rate.

**Table 8:** Feature extraction results.

	CR-Jun	TCR-Jun	CR-Dec	TCR-Dec
1	36.97%	36.97%	40.27%	40.27%
2	22.71%	59.68%	24.21%	64.48%
3	16.31%	75.99%	15.39%	79.87%
4	11.91%	87.90%	10.55%	90.42%
5	9.69%	97.59%	8.13%	98.55%
6	2.41%	100.00%	1.45%	100.00%

According to the results in Table 8, the principal component contribution rate of this research is 95%. Therefore, in the two data sets, the first five principal components are selected as the input of the model.

The training set and test set are then allocated. In the data set from June, 270 groups of data from the first 27 days are used as the training set, and 30 groups from the last three days are used as the test set. In the data set from December, 280 groups of data from the first 28 days are used as training sets, and 30 groups from the last three days are used as test sets.

To validate the effectiveness of PCA, we utilized the MISSA algorithm to predict the sample data before and after feature extraction. The specific results are outlined in Table 9.

**Table 9:** Comparison of prediction results based on PCA.

Month	Model	RMSE	MSE	MAE	Time (ms)
June	PCA-MISSA-SVR	**1.537**	**2.361**	**1.230**	81.7
	MISSA-SVR	1.962	3.851	1.492	107.9
December	PCA-MISSA-SVR	**0.813**	**0.660**	**0.489**	70.9
	MISSA-SVR	1.001	1.002	0.724	98.1

Significant values are in bold.

As shown in Table 9, predictions and prediction times are provided for both datasets before and after feature extraction. It is evident that both the prediction accuracy and time improved after applying PCA compared to the original dataset, thus demonstrating the effectiveness of PCA.

### Data normalization

Because the feature quantity is complex and the numerical difference is too large, to increase the prediction accuracy, all data will be normalized before the prediction and all the data will be normalized to (− 1, 1). The specific formula used is as follows:

12 $$x_i^\prime = 2 \cdot \frac{{{x_i} - {x_{\min }}}}{{{x_{\max }} - {x_{\min }}}} - 1$$

At the same time, to get the correct prediction results, the predicted data will be de-normalized, and the results will be outputted.

### Prediction process of PV power generation based on MISSA-SVM

**Figure 6:**
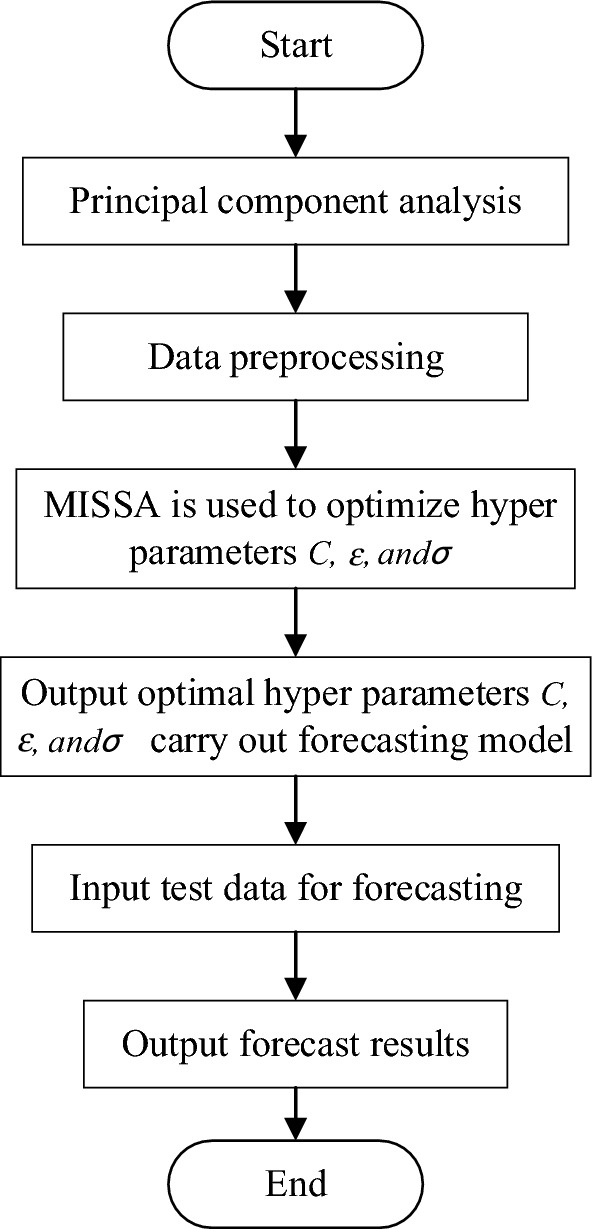
Prediction process based on PCA and MISSA-SVM.

This section elaborates on the specific process of PV power generation prediction based on MISSA-SVM, as shown in Fig. 6.

1. Data pre-processing: Number and classify the two data sets and divides into a training set and a test set. Select the data from the final three days as the test set, and the remaining data as the training set.
2. PCA: According to the standard of 95% of the total contribution rate of the principal components, a PCA is used to process the PV power generation data.
3. Parameter optimization: Hyperparameters, *C*, and* σ* of SVM are optimized with MISSA, and the specific optimization process is shown in pseudo code Algorithm 1.
4. Power generation forecast: Bring the super parameters optimized by MISSA into SVM to establish a prediction model and input the test set for prediction (In Example analysis 1: *C* = 913.934,* σ* = 0.0445. In Example analysis 2: *C* = 868.771, *σ* = 0.001).
5. Result output: The prediction of the PV power generation is completed, and the prediction results are outputted, including the prediction curve, residual curve, RMSE, MSE, MAE, and operation time.

## Prediction of PV power generation based on MISSA-SVM

To verify the performance of the prediction model in this paper, in this section of the proposed model, references^24,38,39^ and some common models are used to predict the power generation and compare the prediction results. The specific models are MISSA-SVM GA-SVM, PSO-SVM, SSA-SVR, and GWO-BPNN. Three evaluation indicators are adopted, namely RMSE, MSE, MAE and R coefficient.

### Example analysis 1

**Figure 7:**
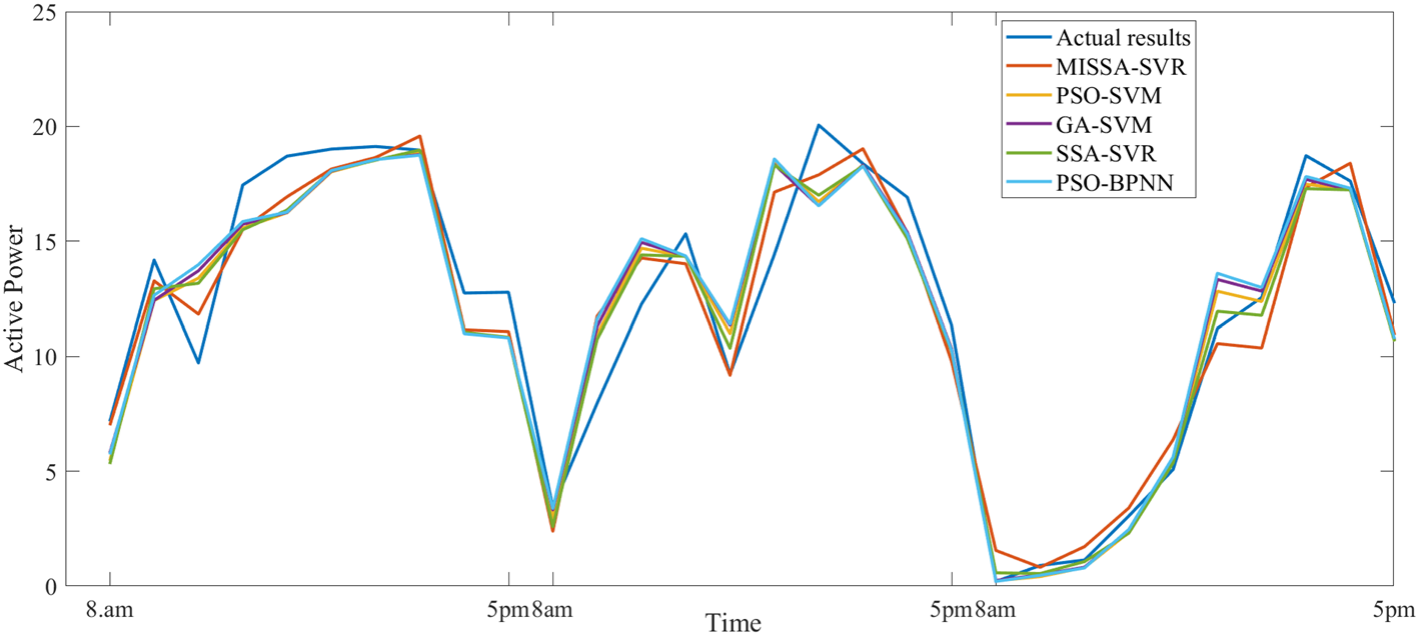
Prediction results based on June data.

**Figure 8:**
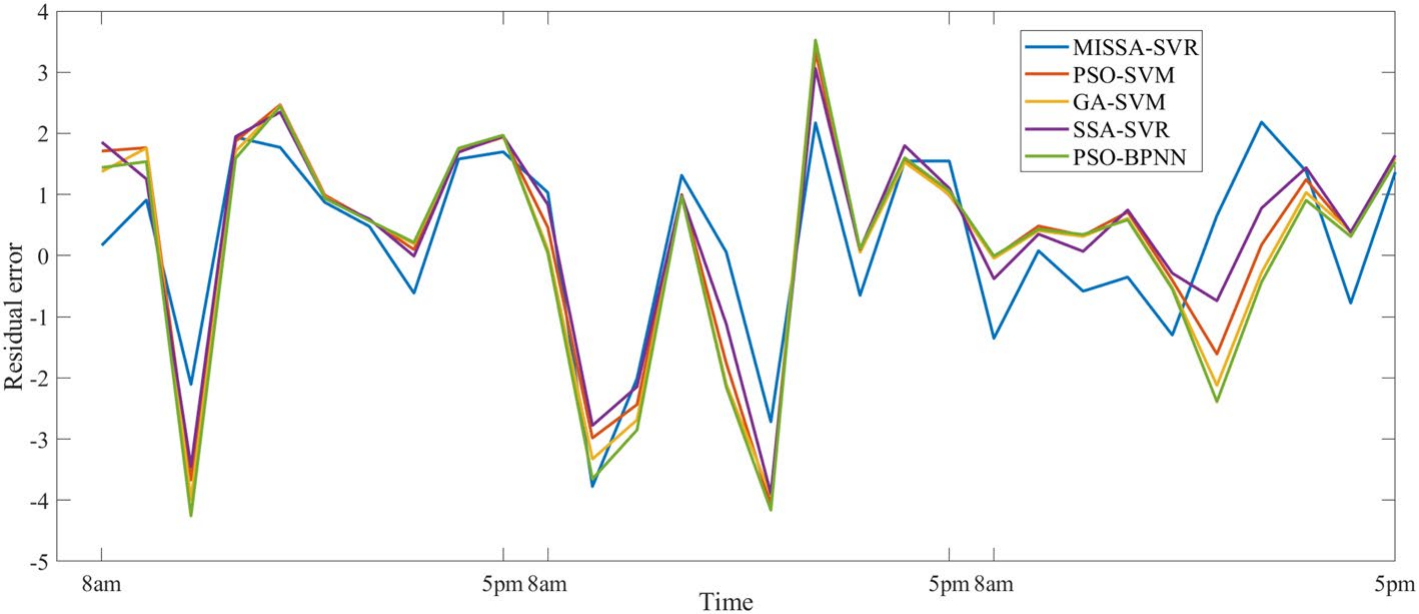
Residual curve based on June data.

**Figure 9:**
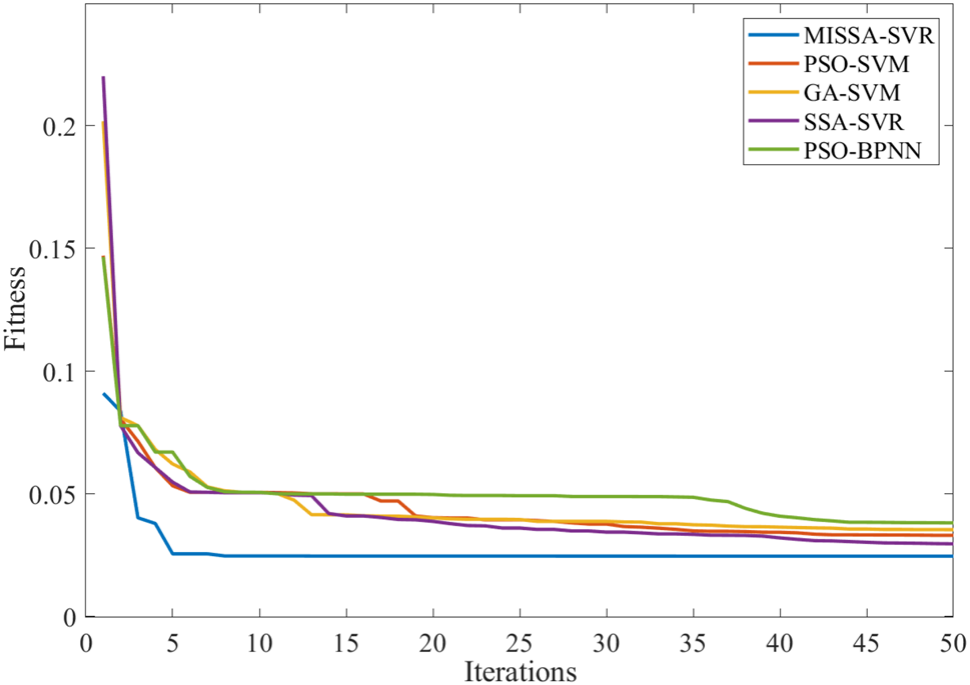
Fitness curve based on June data.

This section analyzes the prediction results of the power generation during the last three days of June, with 30 time periods between 8 a.m. and 5 p.m. The number of iterations of all models is set to 50, and the population number of the algorithm is 30. Table 10 shows three evaluation indicators of the prediction results, Fig. 7 shows the prediction results, Fig. 8 shows the prediction residuals, and Fig. 9 shows the fitness values.

**Table 10:** Prediction results based on June data.

Model	RMSE	MSE	MAE	R coefficient	Time (ms)
MISSA-SVR	**1.537**	**2.361**	**1.230**	**0.9680**	81.7
SSA-SVR	1.668	2.781	1.387	0.9613	76.4
GA-SVM	1.831	3.351	1.433	0.9528	79.1
PSO-SVM	1.783	3.178	1.42	0.9565	83.1
PSO-BPNN	1.921	3.690	1.476	0.9478	213.4

Significant values are in bold.

First, As can be seen from the prediction results in Table 10, compared with the other four models, three evaluation indicators of the proposed model are all the smallest, with the values 1.536, 2.361, and 1.230 respectively. Three evaluation indicators of the other three SVM-based models is larger than that of the proposed model, but the prediction time of this model increased due to the improved methods (3 and 4). Because the PSO model is simple, the prediction time of the PSO-SVM is the lowest; however, the result of prediction of PSO-SVM is the worse. At the same time, PSO-BPNN is analyzed. Because of the complexity of the BPNN model and its dependence on large sample data, the prediction time is the longest and three evaluation indicators of this model is the worst of the five.

Then, analyze Figs. 8 and 9, it can be seen that the prediction curve of the proposed model is the best fit. Compared with the actual results, the prediction results of MISSA-SVM do not have a large prediction error. The prediction results of the other four models are poor, and there is a certain gap between the curve fitting and the prediction results of the model proposed in this paper. The residual curve jitter of the proposed model is also the minimum out of the five.

Furthermore, Fig. 9 shows that the fitness curve of the model in this paper converges the fastest. Because of improved method (1), the initial value of the curve is the best, and the optimal fitness value has been found around the seventh generation.

Finally, by comprehensively analyzing the above prediction results, it can be concluded that compared with the other four prediction models, the prediction performance of the model proposed in this paper has advantages.

It is noting that one data set is used for testing cannot fully prove the effectiveness of the proposed model. Therefore, in “Example analysis 2”, the data in December are predicted and analyzed to further prove the effectiveness and superiority of the proposed model.

### Example analysis 2

**Figure 10:**
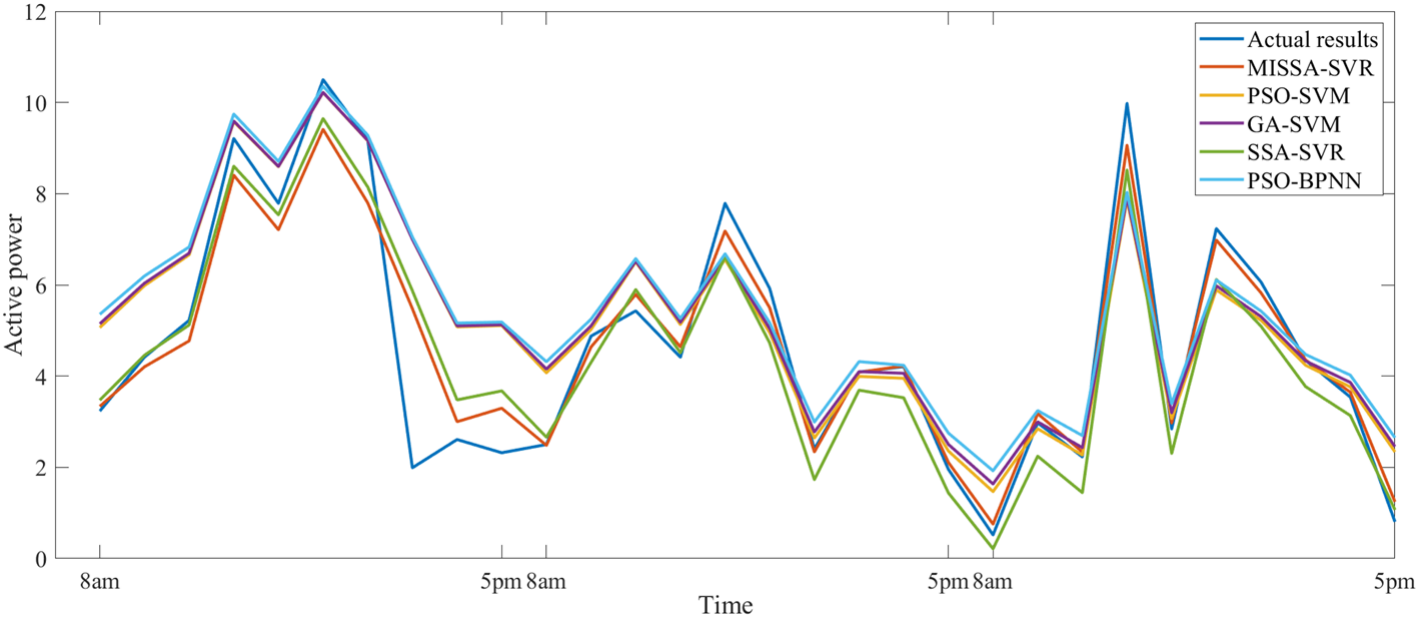
Prediction results based on December data.

**Figure 11:**
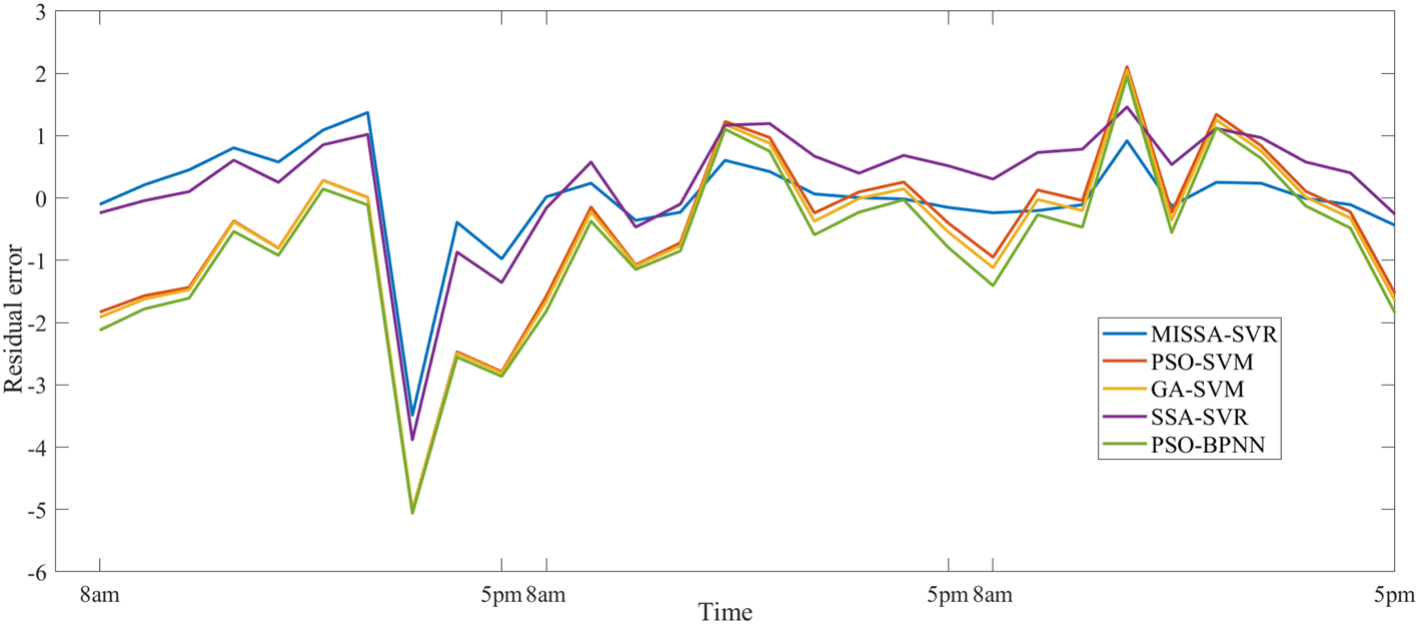
Residual curve based on December data.

**Figure 12:**
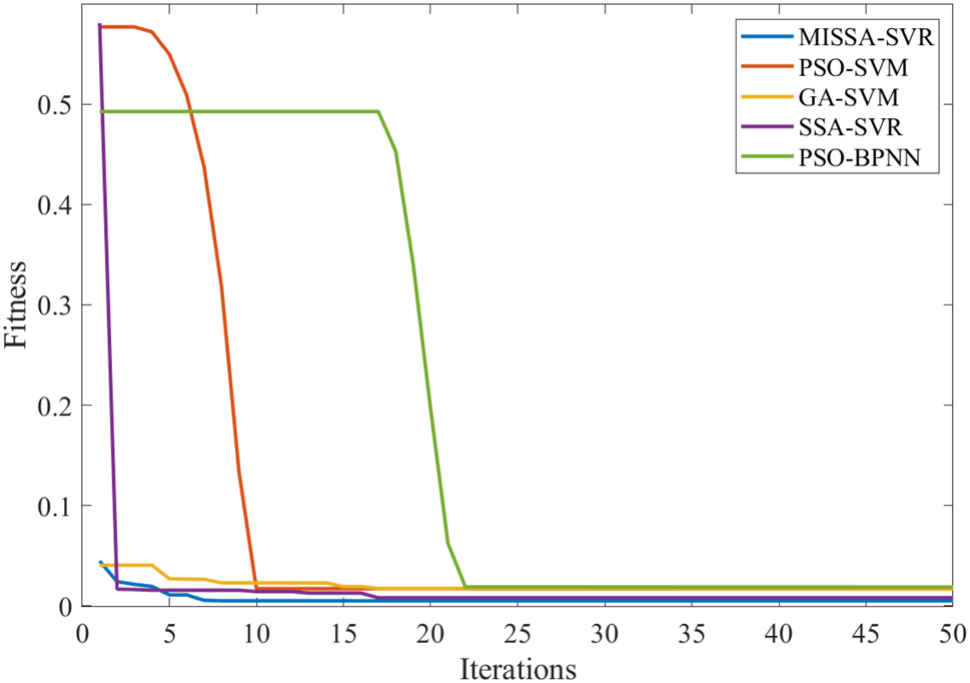
Fitness curve based on December data.

In this section, five models will be used to predict the PV power generation during the last three days of December. The specific data time tag is the same as the parameter setting of the model analyzed in “Example analysis 1”. Table 11 shows three evaluation indicators of the prediction results, Fig. 10 shows the prediction results, Fig. 11 shows the prediction residuals, and Fig. 12 shows the fitness values.

**Table 11:** Prediction results based on December data.

Model	RMSE	MSE	MAE	R coefficient	Time (ms)
MISSA-SVR	**0.813**	**0.660**	**0.489**	0.9625	70.9
SSA-SVR	0.937	0.879	0.635	0.9412	68.1
GA-SVM	1.499	2.247	1.070	0.8489	73.6
PSO-SVM	1.451	2.105	1.010	0.8505	71.3
PSO-BPNN	1.534	2.353	1.122	0.8670	186.3

Significant values are in bold.

First, Table 11 is analyzed. Compared with the other four models, three evaluation indicators of the proposed model are, as was seen with the results of June, all the smallest, with values of 0.813, 0.660, and 0.489, respectively. Three evaluation indicators of the other three SVM-based models are larger than that of the proposed model, but the prediction time of this model is also increased. In comparison with example 1, the result of this example has is the same. Thus, it can be further proved that the prediction performance of the model in this paper is much higher than that of other models.

Then, Figs. 10 and 11 are analyzed. Compared with the actual results, the prediction results of MISSA-SVM only produced a large prediction error in the afternoon of the first day, and the prediction curve in other periods has a good fit. The prediction results of the other four models are poor and there is a certain gap between the curve fitting and the prediction results of the model proposed in this paper.

As shown Fig. 12, the fitness curve of the model proposed in this paper began to converge in the seventh generation, and compared with the other four models, the fitness value found is superior. In addition, the SSA-SVM found a better fitness value, but its curve began to converge only in the 17th generation.

Finally, according to Table 11, three evaluation indicators of the SSA-SVM has certain advantages, but it also takes a long time to predict because of the complexity of the optimization strategy of the SSA.

At the same time, it can be seen that in the SVM-based model, the prediction time is further increased due to the improved methods (3 and 4), but the three evaluation indicators obtained has significant advantages. By comprehensively analyzing the prediction results, MISSA-SVM can generate a more accurate prediction at the expense of a small part of the prediction time.

## Conclusion

In order to improve the accuracy of the power generation prediction of PV power stations, a prediction model based on SVR is proposed. First, a PCA is used to analyze the power generation data and to propose the principal components with low correlation. Second, four improved methods are proposed based on tent chaos initialization, nonlinear attenuation of predator probability, chaos-based dynamic reverse learning strategy, and selection strategy to improve the SSA. Finally, the PV power generation prediction model based on the MISSA-SVR is established. The conclusions of this paper are as follows:

1. Six benchmark functions were used to test the optimization performance of the MISSA’s other four metaheuristic algorithms. The test results effectively proved that the MISSA has the fastest optimization speed and the highest precision, which can ensure the accuracy of the MISSA in SVR super parameter optimization.
2. After using two kinds of data sets for example analysis, compared with the other prediction models, the MISSA-SVR greatly improved prediction accuracy at the expense of some prediction efficiency. The three evaluation indicators obtained by simulation are optimal, which proves the effectiveness of the prediction model proposed in this paper.

To sum up, the multi-strategy improvement method proposed in this paper has a high reference value. At the same time, the proposed prediction model has a relatively excellent prediction effect, and it can also be applied to most prediction problems in related fields.

The methods proposed are only applicable to small-sample, low-dimensional photovoltaic power generation data. Future research will focus on validating these methods using online prediction techniques based on deep learning. Additionally, efforts will be made to develop prediction models suitable for various types of data by integrating the proposed methods.

## Data Availability

The datasets used during the current study available from the corresponding author on reasonable request.
